# Bacteria can be selected to help beneficial plasmids spread

**DOI:** 10.1371/journal.pbio.3001489

**Published:** 2021-12-21

**Authors:** Tatiana Dimitriu, Dusan Misevic, Ariel B. Lindner, Francois Taddei, Sam P. Brown

**Affiliations:** 1 Environment and Sustainability Institute, Biosciences, University of Exeter, Penryn, United Kingdom; 2 Université de Paris, INSERM U1284, Center for Research and Interdisciplinarity, Paris, France; 3 School of Biological Sciences, Georgia Institute of Technology, Atlanta, Georgia, United States of America; 4 Center for Microbial Dynamics and Infection, Georgia Institute of Technology, Atlanta, Georgia, United States of America

## Abstract

A recent commentary raised concerns about aspects of the model and assumptions used in a previous study which demonstrated that selection can favor chromosomal alleles that confer higher plasmid donation rates. Here, the authors of that previous study respond to the concerns raised.

In our original work [1], we demonstrated experimentally that selection can favor chromosomal alleles that confer higher plasmid donation rates, given the plasmid is beneficial and the recipient has an elevated chance of carrying the donor allele (i.e., preferential donation to kin). Our experiments demonstrated this effect via 2 mechanisms of preferential donation: biased conjugation rates and structured populations. We interpreted these results through the lens of kin selection theory (benefits via horizontal gene transfer to kin), supported by simulations and an analytical fitness function model. These results hold importance by outlining that the evolution of plasmid transfer rates (a key aspect of the antibiotic resistance crisis) is not necessarily the sole product of selection on the plasmid itself and forms part of a broader series of papers from our labs investigating the sociomicrobiology of plasmids [2–4].

A new commentary raises concerns over our fitness function model, flagging issues with both the structure of the model and assumptions made in our analysis [5]. We stand by the general conclusions of our work but accept that our fitness function and stated analysis assumptions could be better formulated. Our initial fitness function is heuristic in the sense it was designed to capture general processes acting on the fitness of individuals, dependent on the plasmid and donor allele status—without explicitly modeling the myriad demographic events of dispersal, reproduction, conjugation, and death that result in selective shifts across a metapopulation of cells. Specifically, we captured the “force of infection” faced by an uninfected cell as the product of average plasmid prevalence and average donor allele prevalence in the local patch (*p*_*j*_*q*_*j*_; see commentary for notation details). We agree with the authors that this force of infection is better phrased as the average of the product ((1/*N*)∑*p_ij_ q_ij_*), in part because this avoids the potential pathology under limit conditions described by the authors, but also because this approach better highlights that the particular social trait in question is an “other only” cooperative trait [6], illustrated by commentary equation [2], where transmission to self and transmission to others are separated. This separation has the important consequence of highlighting that unlike many microbial social traits where benefits accrue to a group (including self), a cooperative plasmid donor trait can only benefit other cells that lack the plasmid. Given established costs of donation (e.g., see figure S2 in our original article), this defines our “donor” behavior as an altruistic trait, which can, therefore, only be favored by selection given nonrandom interactions among individuals (e.g., [7]).

Our experimental results outline 2 mechanisms of nonrandom interactions: preferential donation to kin and population structure. Each of these mechanisms will generate positive covariances between focal individual *q*_*ij*_ and non-self-recipient *q*_*j*_ donor allele states (cov(*q*_*j*_, *q*_*ij*_*) >* 0). The pathway via preferential donation to kin (order-of-magnitude differences according to our analyses and more recent measurements among lineages coexisting within natural populations [8]) will also likely generate positive covariances between donor and recipient abilities (cov(*s*_*ij*_, *q*_*ij*_) > 0). In contrast, to arrive at the result that selection always works against plasmid donor alleles (equation [4]), the commentary makes the assumption that both of the above covariances are zero. We suggest that the additional analyses begun by the authors are an exciting starting point to better map selection on donor alleles, under a broader array of defined assumptions on cell–cell and gene–gene structure, ideally informed by data on structures found in natural bacterial populations.
